# Tsallis *q*-Statistics Fingerprints in Precipitation Data across Sicily

**DOI:** 10.3390/e26080623

**Published:** 2024-07-24

**Authors:** Vera Pecorino, Alessandro Pluchino, Andrea Rapisarda

**Affiliations:** 1 Dipartimento di Fisica e Astronomia “Ettore Majorana”, Università di Catania, 95123 Catania, Italy; pecov1800@gmail.com (V.P.); andrea.rapisarda@ct.infn.it (A.R.); 2 INFN Sezione di Catania, 95123 Catania, Italy; 3 Complexity Science Hub, 1080 Vienna, Austria

**Keywords:** Tsallis q-statistics, Sicily rainfall data, climate change

## Abstract

Precipitation patterns are critical for understanding the hydrological and climatological dynamics of any region. Sicily, the largest island in the Mediterranean sea, with its diverse topography and climatic conditions, serves as an ideal case study for analyzing precipitation data, to gain insights into regional water resources, agricultural productivity, and climate change impacts. This paper employs advanced statistical physics methods, particularly Tsallis *q*-statistics, to analyze sub-hourly precipitation data from 2002 to 2023, provided by the Sicilian Agrometeorological Informative System (SIAS). We investigate several critical variables related to rainfall events, including duration, depth, maximum record, and inter-event time. The study spans two decades (2002–2012 and 2013–2023), analyzing the distributions of relevant variables. Additionally, we examine the simple returns of these variables to identify significant temporal changes, fitting these returns with q-Gaussian distributions. Our findings reveal the scale-invariant nature of precipitation events, the presence of long-range interactions, and memory effects, characteristic of complex environmental processes.

## 1. Introduction

Precipitation patterns play a crucial role in understanding the hydrological and climatological dynamics of any region. In the context of Sicily, an island characterized by diverse topographical and climatic conditions, analyzing precipitation data provides valuable insights into regional water resources, agricultural productivity, and climate change impacts. Being the largest island in the Mediterranean sea and located in the middle of it, Sicily is also of great interest for the entire Mediterranean area.

A robust statistical approach is essential for uncovering the underlying patterns and anomalies in precipitation data, thereby enabling more accurate predictions and effective water management strategies. In recent years, the application of advanced statistical physics methods has provided new insights into the analysis of complex environmental data [1,2,3]. In particular, various studies have emphasized the importance of different probability distributions in rainfall analysis. For instance, the Poisson Hurwitz–Lerch zeta distribution has been used to model the frequency of interarrival times and rainfall depths [4]. Some studies assumed that the daily precipitation intensities are distributed according to a Gamma [5] or a mixed exponential [6], light-tailed or heavy tailed distributions [7,8], while other authors found a log-normal [9] or a stretched exponential [10] distribution. Probability distributions of daily rainfall extremes have also been studied to make rainfall inferences [11], and entropy-based derivations of probability distributions have been applied to daily rainfall data [12]. Understanding the fundamental probability distribution for heavy rainfall can provide insights into extreme weather events [10], and analyzing extreme rainfall trends is crucial for evaluating depth–duration–frequency curves in climate change scenarios [13].

In this context, *q*-statistics offer a robust framework for analyzing the variability and distribution of complex environmental data, as they are particularly effective in capturing the non-linear and multi-scalar nature of such events. Raw data often follow power law distributions, indicating the presence of scale-invariant processes and the frequent occurrence of extreme events [14]. For instance, Yang et al. demonstrated the power-law behavior of hourly precipitation intensity and dry spell duration over the United States, highlighting the scale-invariant nature of these phenomena [15]. Additionally, studies have focused on the use of probability distributions in rainfall analysis [16], and memory in volatility return intervals and a decumulative probability function, following the methodologies usually employed in the study of financial markets [17]. Decumulated data can be effectively modeled using Tsallis exponential distributions, which account for long-range interactions and memory effects typical of many natural processes. Pluchino et al. showed the applicability of Tsallis statistics in capturing long-term correlations at the edge of chaos [18]. Similarly, Ludescher et al. described the universal behavior of interoccurrence times between losses in financial markets using Tsallis statistics [19], emphasizing the presence of memory effects [20]. Furthermore, the simple returns of these events, representing changes over time, conform to q-Gaussian distributions [21], which better capture the heavy tails and non-Gaussian behavior observed in the data. Recently, Tsallis statistics were also successfully applied by Greco et al. to study acoustic emissions close to the rupture point of compressed rocks of various natures [22,23]. Bogachev and Bunde (2008) discussed memory effects in the statistics of interoccurrence times between large returns in financial markets, demonstrating the relevance of q-Gaussian distributions in modeling heavy tails and non-Gaussian behaviors [24]. Yamasaki et al. also highlighted scaling and memory in volatility return intervals in financial markets, further supporting the use of q-Gaussian distributions for this kind of analysis [25].

In this paper, we present a comprehensive analysis of precipitation data through the lens of *q*-statistics. Specifically, we analyzed sub-hourly precipitation data from 2002 to 2023, provided by the Sicilian Agrometeorological Informative System (SIAS). The considered dataset comprises records from 107 meteorological stations, with a focus on nine key rain gauges located in Messina, Catania, Siracusa, Ragusa, Enna, Caltanissetta, Agrigento, Trapani, and Palermo. We examined several key variables related to rainfall events, including

- Duration [minutes], the length of consecutive wet records;
- Depth [mm], the total amount of precipitation during an event;
- Maximum record [mm/10′], the highest recorded precipitation in a 10 min interval during an event.

To investigate the temporal evolution of these variables, we analyzed their distributions over two decades (2002–2012 and 2013–2023). We also explored simple returns of these variables. In order to characterize our distributions and to identify any significant changes over time, we considered Tsallis q-statistics [19].

Our analysis aimed to uncover patterns and trends in Sicilian precipitation data, providing insights into regional climate dynamics and potential impacts of climate change. This study could offer valuable information to scientists, policymakers, and stakeholders involved in environmental and water resource management in Sicily.

## 2. Dataset and Relevant Variables

**Figure 1:**
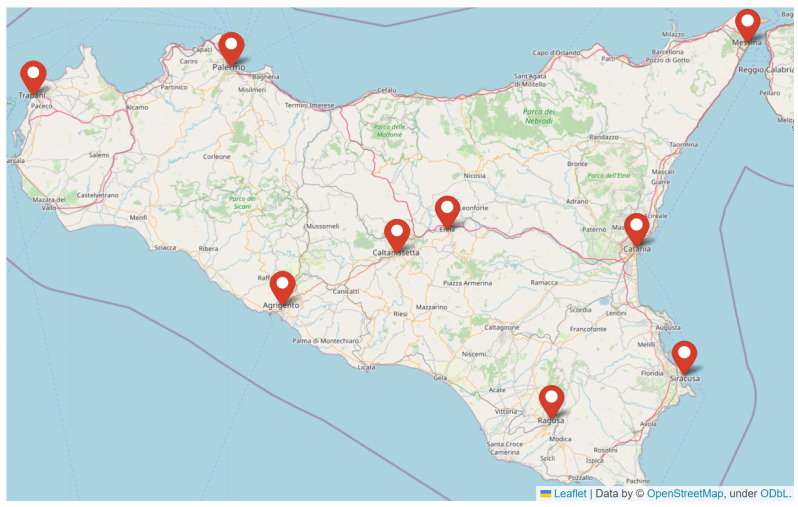
SIAS meteorological network: location of the nine rain gauge stations considered in this paper. See text for more details.

This study is based on precipitation records from 2002 to 2023 provided by a robust and extensive rain gauge network under the maintenance of the Sicilian Agrometeorological Informative System (SIAS), which comprises 107 meteorological stations. We included in our study the rain gauges of Messina, Catania, Siracusa, Ragusa, Agrigento, Trapani, and Palermo, which are the most populated cities on the coastline (Istat—Statistical National Insitute—report 2018/2019). In order to include the midland area, we added two more cities, namely Enna and Caltanissetta. We analyzed precipitation time series with a 10 min basis across the nine selected rain gauge stations, see Figure 1. The minimum quantity observable with the SIAS’s pluviometers was 0.2 mm and, as we mentioned, the time resolution was 10 min; we used such granular data per station and built a new time series based on rainfall events.

A rainfall event over a rain gauge in our dataset is an episode of consecutive wet records, i.e., the consecutive not null rows. It follows that each rainfall event can be characterized by two quantities: a duration [in minutes] and a depth [in mm]. The duration of an event is the length of consecutive wet records or, in other words, the number of consecutive not null rows. The rainfall depth relative to an event is the sum of precipitation amount over the corresponding event duration, in other words, how much it rained during the event. Each rainfall event is formed by one or more not null records, and one of those values is the maximum value recorded during the event. We focused our study on the previous relevant variables related to rainfall events: depth [mm], maximum record [mm/10′], and duration [minutes]. The first two variables are related to the amount of precipitation, whilst the duration is a temporal variable. We grouped results following such criteria. As the whole dataset covers a time span of 22 years, we arbitrarily chose to perform our analysis with a 11-year scale. We adopted a seasonal approach exploring the principal features of the distributions of these variables, in order to evaluate the presence of certain temporal trends across decades.

## 3. Statistical Analysis of Precipitation Events

### 3.1. Probability Density Functions

First, we analyzed the probability density function (PDF) for our relevant variables, considering both seasonal and decade variations. This approach helps in managing the complexity and volume of the data, while still providing clear insights into the overall trends.

**Figure 2:**
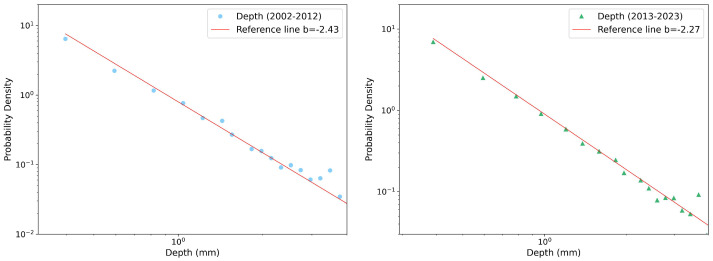
Probability density function of autumn rainfall depth in log–log scale and its fits with a power law (red line) for the two decades considered: 2002–2012 (**left** panel) and 2013–2023 (**right** panel). The slopes of the fits are also reported, see text for more details.

**Figure 3:**
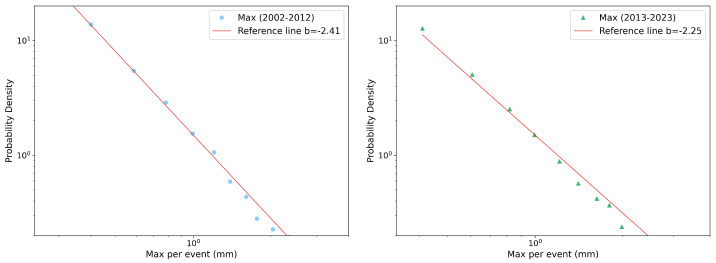
Probability density function of autumn max per event in log–log scale and the fit with a power law (red line) for the two decades considered: 2002–2012 (**left** panel) and 2013–2023 (**right** panel). The slopes of the fits are also reported, see text for more details.

**Figure 4:**
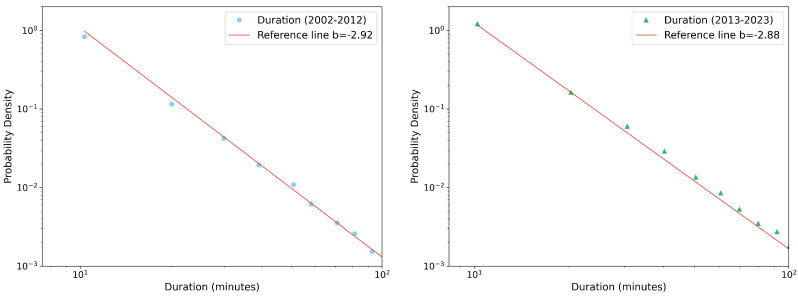
Probability density function of autumn rainfall event duration in log–log scale and its fits with a power law (red line) for the two decades considered: 2002–2012 (**left** panel) and 2013–2023 (**right** panel). The slopes of the fits are also reported, see text for more details.

Due to the extensive number of reports generated for each season, variable, and decade, we present only a few selected plots, then we can summarize all the results in a more compact way. Figure 2, Figure 3 and Figure 4 illustrate PDFs of the events’ rainfall depth, maximum record, and duration, respectively, cumulated over all the gauge stations for the autumn season in each of the two decades, 2002–2012 (left panel) and 2013–2023 (right panel). All the distributions can be well fitted by power-law functions $y∼x^{-b}$. Performing a $\chi^{2}$ test, the power-law fit always resulted in a *p*-value < 0.05, indicating the scale-invariant nature of the precipitation data, but with different slopes (reported in the legends).

**Figure 5:**
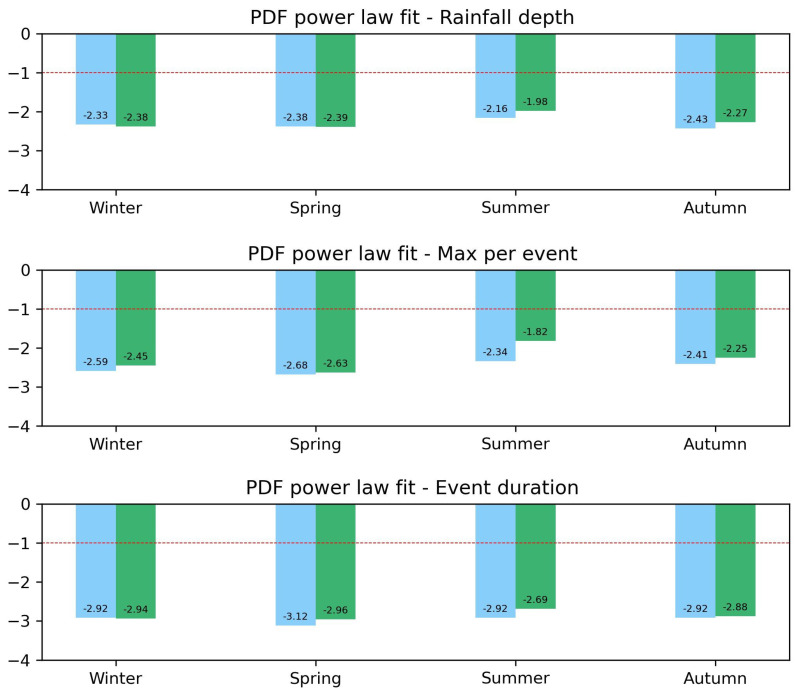
We report the values of the slopes of the power-law fits for the events’ rainfall depth (**top** panel), maximum record (**central** panel), and duration (**bottom** panel). The different colors refer to the two decades studied: blue for the period 2002–2012 and green for the period 2013–2023. Differences between the two decades can be appreciated, in particular for summer. An horizontal red dotted line has been added as reference for the eye. See text for more details.

In Figure 5, we compare, as bar charts, the slopes of all the power-law fits performed on the seasonal distributions of the same three variables for the two considered decades. Bars are colored in blue for 2002–2012 and in green for 2013–2023. The analysis of rainfall depth and maximum record (top and central panel, respectively) revealed a sensitive increase in extreme events, indicated by a lower absolute value of the slopes, only for summer and autumn of the second decade. A slight increase in the events’ duration for the second decade can also be appreciated (bottom panel), but only for spring and summer. The winter behavior remained largely unchanged from one decade to another, even if a small decrease in rainfall depth together with a slight increase in the max intensity are visible for this season (in the top and central panels, respectively).

The observed changes in the slopes over the decades indicate a possible increase in the frequency and intensity of extreme precipitation events, which might be attributed to changing climate patterns affecting atmospheric turbulence and energy distribution.

### 3.2. Decumulative Probability Distributions

**Figure 6:**
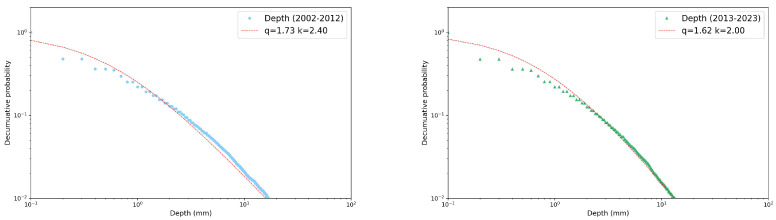
Decumulative probability distributions in log–log scale of winter rainfall depth per event and their *q*-exponential fits. Comparison between decades: 2002–2012 (**left** panel) and 2013–2023 (**right** panel).

**Figure 7:**
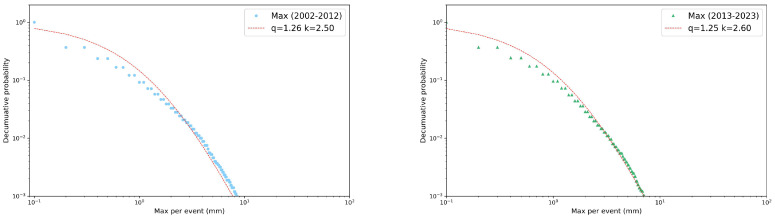
Decumulative probability distributions in log–log scale of winter maximum per event and their *q*-exponential fits. Comparison between decades: 2002–2012 (**left** panel) and 2013–2023 (**right** panel).

**Figure 8:**
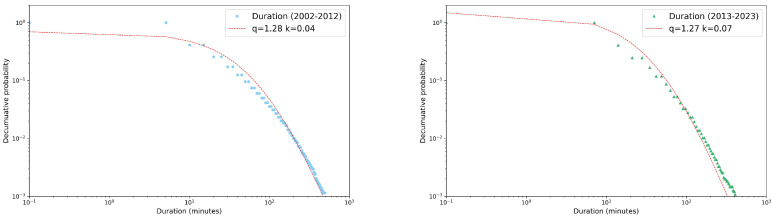
Decumulative probability distributions in log–log scale of winter event duration and their *q*-exponential fits. Comparison between decades: 2002–2012 (**left** panel) and 2013–2023 (**right** panel).

In this section, we investigate the decumulative probability distributions for our three relevant variables in the four seasons and the two decades by means of *q*-statistics. For each variable, we plot the fraction of precipitation events (collected for all the gauge stations), with values above the threshold reported on the *x*-axis. As in the previous section, we start by presenting some selected examples of these distributions in the two decades. In particular, in Figure 6 and Figure 7 we analyzed the rainfall depth and the maximum record in winter, while in Figure 8 we focused on the event duration in summer. All the distributions resulted as well fitted by Tsallis *q*-exponential functions in the usual form [19]:

(1) $$e_{q}\left(x\right)={\left[1+(1-q)kx\right]}^{\frac{1}{1-q}},$$

where *q* is the entropic index and *k* is a constant that controls the inflection point of the curve. For q=1, the standard exponential function is recovered. Values of the entropic index greater than 1 indicate fat tailed tails and typically quantify the degree of long-range correlations and memory effects present in the system, expressed by the entity of the deviation from unit. Applying the $\chi^{2}$ test, the Tsallis *q*-exponential fit consistently yields a *p*-value lower than 0.05. In these examples, the entropic index shows a slight difference between the two decades only for winter rainfall depth, while the winter maximum intensity and summer duration remained largely unchanged.

The use of Tsallis *q*-statistics with *q* > 1 indicated that the precipitation events exhibited long-range correlations and memory effects, deviating from classical exponential behavior. This is consistent with systems that have persistent interactions over time, suggesting that atmospheric processes have significant temporal dependencies.

**Figure 9:**
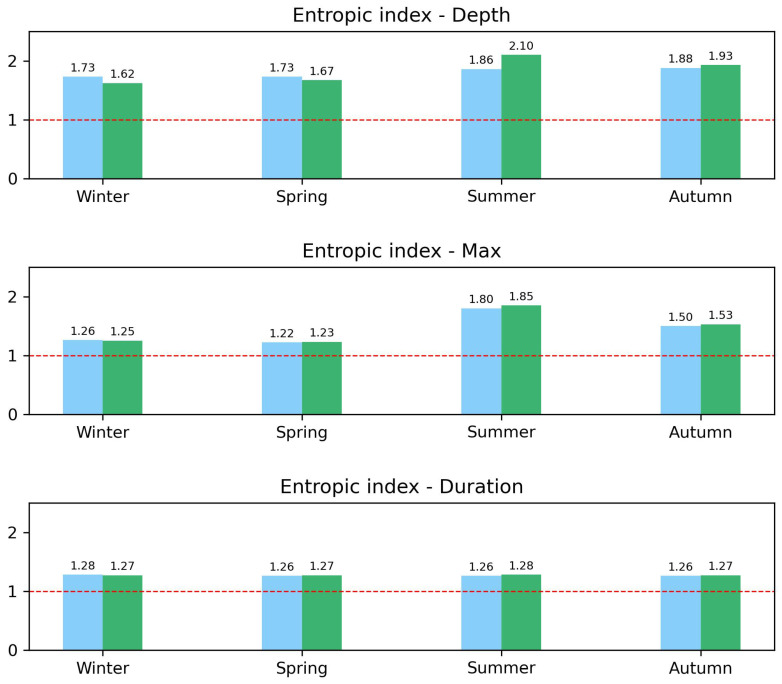
Seasonal bar chart of the entropic index *q* calculated for the rainfall depth, maximum intensity per event, and duration decumulative distributions. Comparison between decades: 2002–2012 (blue) and 2013–2023 (green). A red dotted line as be added as reference for q=1. See text for more details.

Detailed results of the values of the entropic index *q* for each variable and each season are reported in the three panels of Figure 9, where bar charts are again colored in blue and green for 2002–2012 and 2013–2023, respectively.

The changes in the entropic index *q* across different decades and seasons imply variations in the degree of correlations and memory effects within the atmospheric system. This may be indicative of evolving climatic conditions and their impact on the statistical properties of precipitation events.

In the top panel, the comparison of entropic indexes for the events’ rainfall depth revealed an increase in correlations in the second decade for summer and autumn only, while a decrease was observed for winter and spring. A slight increase in the index *q* among decades can also be observed only in summer for the maximum per event intensity (central panel). All the other comparisons in both the central and bottom panels only show very similar values for the entropic index.

The observed variations in the entropic index *q* for different seasons and decades suggest that the degree of correlation and memory in precipitation events has changed over time, potentially due to climatic changes. Increases in *q* indicate stronger correlations and memory effects, particularly in summer and autumn, reflecting changes in atmospheric dynamics.

### 3.3. Returns Distribution

Finally, in this section, we investigate the behavior of simple returns distributions for each relevant variable, for the different seasons and decades studied. We consider normalized simple returns *R* defined as follows:

(2) $$R=\frac{\left[\left(x_{n+1}-x_{n}\right)-x_{mean}\right]}{\sigma_{std.dev}}.$$

**Figure 10:**
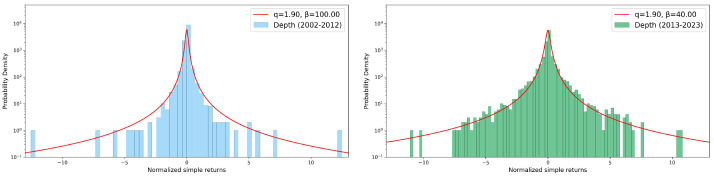
Simple returns in log–lin scale: data and *q*-Gaussian fits of spring events’ rainfall depth. The comparison between the two considered decades, i.e., 2002–2012 (**left** panel) and 2013–2023 (**right** panel), does not show any relevant differences in the entropic index *q*.

**Figure 11:**
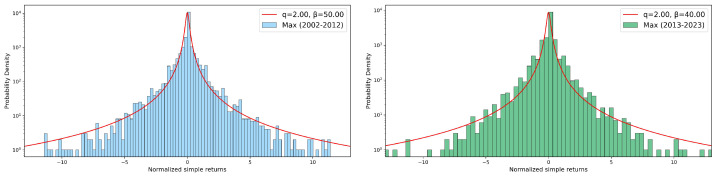
Simple returns in log–lin scale: data and *q*-Gaussian fits of winter maximum per event. In the **left** panel, we report the 2002–2012 decade, while the **right** panel shows the 2013–2023 decade. See text for more details.

**Figure 12:**
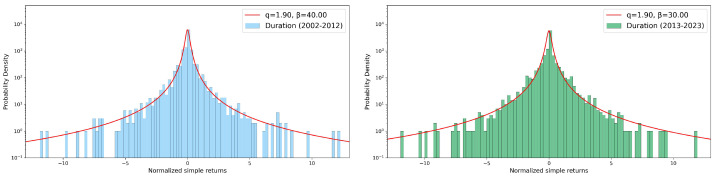
Simple returns in log–lin scale: data and *q*-Gaussian fits of spring rainfall event duration. In the **left** panel we report the 2002–2012 decade, while the **right** panel shows the 2013–2023 decade. See text for more details.

**Figure 13:**
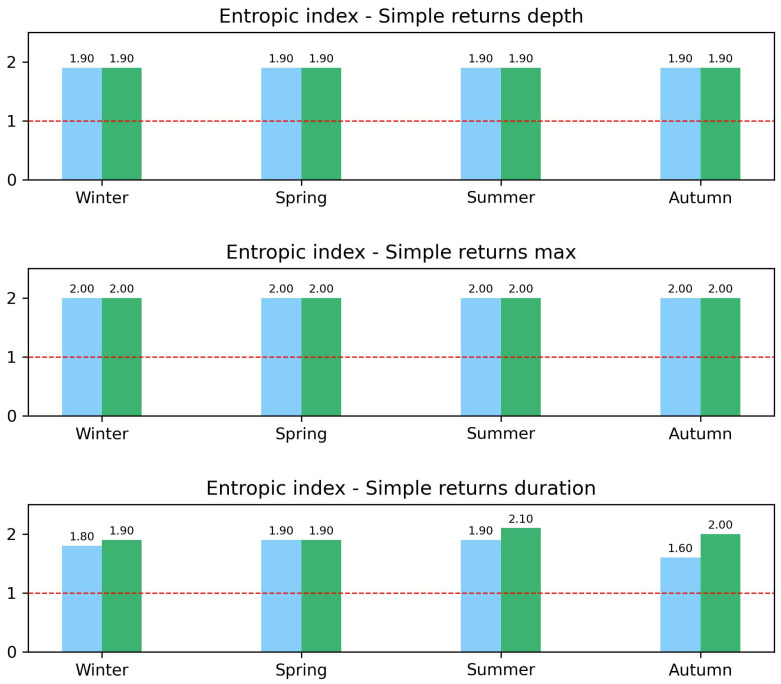
Bar chart of entropic index *q* for rainfall depth (**top** panel), maximum intensity recorded per event (**central** panel), and event duration (**bottom** panel) are reported for decade (2002–2012 in green and 2013–2023 in blue) and season. A red dotted line as be added as reference for q=1. See text for more details.

With the distributions of returns being symmetric, they are well fitted by Tsallis *q*-Gaussian curves defined as [19]:

(3) $$G_{q}\left(x\right)=A{\left[1-\left(1-q\right)\beta x^{2}\right]}^{\frac{1}{1-q}},$$

where *A* is a normalization parameter, *q* is the entropic index, and β is a parameter related to the spread around the mean. Values of entropic index greater than unit quantify deviations from a Gaussian behavior, also indicating a violation of the standard central limit theorem due to correlations present in the system. In Figure 10, Figure 11 and Figure 12, we report, as in the previous sections, some seasonal examples of distributions of simple returns for our three variables, comparing the two decades: the spring season was chosen for both rainfall depth and duration, with the winter season for the maximum recorded value. No relevant differences among decades are visible in any case for the entropic index *q*, although different values of β were obtained. Such an absence of any change in *q* during 2002–2012 and 2013–2023 can be also appreciated in the summary presented in Figure 13, where we report the bar charts of the entropic index values extracted from *q*-Gaussian fits. For both rainfall depth (top panel) and maximum intensity (central panel), the fitted entropic index does not vary significantly across decades and seasons, suggesting a consistent statistical behavior over time. However, the event duration (bottom panel) shows a substantial increase in winter and, in particular, in summer and autumn, indicating potential changes in the dynamics of rainfall events in these seasons over the considered decades.

The $\chi^{2}$ test of fitting of simple returns with Tsallis *q*-Gaussian curves yielded a *p*-value <0.05. We observe that the exponent *q* is always greater than one, indicating the presence of correlations and deviations from the Gaussian distribution. This suggests that the precipitation events exhibit complex dynamics and memory effects, not fully captured by traditional Gaussian statistics. The lack of significant changes in *q* across decades for rainfall depth and maximum intensity suggests that these aspects of precipitation events have remained statistically stable. However, the increase in *q* for event duration indicates evolving dynamics in how long precipitation events last, potentially reflecting changes in atmospheric conditions over time.

These results highlight the complexity and evolving nature of precipitation event dynamics. The consistent behavior in the entropic index *q* for rainfall depth and maximum intensity suggests a stable underlying process, whereas the increase in *q* for event duration points to changes in how precipitation events are temporally distributed, possibly due to shifts in atmospheric dynamics or climate change.

## 4. Discussion and Conclusions

In this study, we analyzed sub-hourly precipitation data from nine rain gauge stations located in the main cities of Sicily over two decades, 2002–2012 and 2013–2023. Our analysis focused on several key features of rainfall events: depth, maximum recorded value of the event, and duration of the event. The aim was to understand the statistical properties of these variables and possible changes over time.

Our analysis provided, for the first time, a comprehensive quantitative analysis of precipitation data across Sicily using Tsallis *q*-statistics, revealing significant insights into the statistical properties of rainfall events. The power law distributions of the relevant variables suggest the presence of scale-invariant behavior: in fluid dynamics, this points to the influence of turbulence and fractal-like atmospheric phenomena. The *q*-exponential and *q*-Gaussian distributions highlight the presence of long-range correlations and memory effects in the data, suggesting that atmospheric processes are influenced by persistent interactions over time.

The application of Tsallis *q*-statistics in this context has proven to be particularly valuable. By comparing the deviation from exponential and Gaussian behavior among decades and seasons, we were able to capture the deep out-of-equilibrium nature of the precipitation data, which classical statistics often fail to describe accurately. Tsallis *q*-statistics, with a foundation in non-extensive entropy, provide a more flexible and encompassing framework that accounts for the complex dynamics and interactions inherent in environmental data. This non-extensive behavior indicates that precipitation events have significant long-range dependencies and correlations, reflecting the complex, interconnected nature of atmospheric dynamics.

Our findings indicate, in several cases, notable changes among the two decades considered, particularly during the summer and—to a lesser extent—the autumn seasons. We observed an increase in correlations, on one hand, in the decumulative distributions for rainfall depth and the maximum intensity of events and, on the other hand, in the normalized returns distributions for the event duration. This increase in correlations and memory effects, as indicated by the higher entropic index *q*, suggests that the precipitation system has become more interconnected and influenced by long-term climatic factors, which could be a result of ongoing climate change. These quantitative changes, if correctly interpreted, could have significant implications for water resource management and agricultural planning in Sicily, especially in the context of climate change adaptation. In fact, the investigation of the underlying mechanisms driving the observed changes in rainfall patterns could offer valuable insights for developing adaptive strategies to mitigate the impacts of climate variability and change.

By providing a straightforward and accessible approach, q-statistics offer valuable insights into complex hydrometeorological processes, especially in regions with prevalent non-equilibrium conditions like the Mediterranean.

Overall, this study contributes to the growing body of knowledge on precipitation variability and its impacts, offering a valuable resource for scientists, policymakers, and stakeholders involved in environmental and water resource management in Sicily. The application of q-statistics reveals richer structures and long-range dependencies in precipitation data, aiding in better risk analysis, modeling, and decision support.The use of Tsallis *q*-statistics provides a robust tool for understanding the complexity of precipitation patterns and their broader climatic implications. By revealing the non-Gaussian nature and the long-range correlations in precipitation data, this approach helps in better modeling and predicting extreme weather events, which are crucial for effective climate change adaptation strategies. Future research should focus on extending this analysis to other regions and incorporating additional climatic variables, to provide a more detailed understanding of precipitation dynamics.

## Data Availability

Data are available on request.
